# Evidence for natural antisense transcript-mediated inhibition of microRNA function

**DOI:** 10.1186/gb-2010-11-5-r56

**Published:** 2010-05-27

**Authors:** Mohammad Ali Faghihi, Ming Zhang, Jia Huang, Farzaneh Modarresi, Marcel P Van der Brug, Michael A Nalls, Mark R Cookson, Georges St-Laurent, Claes Wahlestedt

**Affiliations:** 1 Department of Neuroscience, The Scripps Research Institute, Scripps Florida, 130 Scripps Way, Jupiter, FL 33458, USA; 2 Department of Molecular Therapeutics, The Scripps Research Institute, Scripps Florida, 130 Scripps Way, Jupiter, FL 33458, USA; 3 T-6, Los Alamos National Laboratory Los Alamos National Laboratory, Los Alamos, NM 87545, USA; 4 Center for Nonlinear Studies, Los Alamos National Laboratory, Los Alamos, NM 87545, USA; 5 Miami Institute for Human Genomics, Miller School of Medicine, 1501 NW 10th Ave, Miami, FL 33101, USA; 6 Laboratory of Neurogenetics, Intramural Research Program, National Institute on Aging, NIH, Bldg 35, 9000 Rockville Pike, Bethesda, MA 20892, USA; 7 Department of Biology, Brown University, 244 Thayer Street, Providence, RI 02912, USA

## Abstract

**Background:**

MicroRNAs (miRNAs) have the potential to regulate diverse sets of mRNA targets. In addition, mammalian genomes contain numerous natural antisense transcripts, most of which appear to be non-protein-coding RNAs (ncRNAs). We have recently identified and characterized a highly conserved non-coding antisense transcript for beta-secretase-1 (*BACE1*), a critical enzyme in Alzheimer's disease pathophysiology. The *BACE1*-antisense transcript is markedly up-regulated in brain samples from Alzheimer's disease patients and promotes the stability of the (sense) *BACE1 *transcript.

**Results:**

We report here that *BACE1*-antisense prevents miRNA-induced repression of *BACE1 *mRNA by masking the binding site for miR-485-5p. Indeed, miR-485-5p and *BACE1*-antisense compete for binding within the same region in the open reading frame of the *BACE1 *mRNA. We observed opposing effects of *BACE1*-antisense and miR-485-5p on BACE1 protein *in vitro *and showed that Locked Nucleic Acid-antimiR mediated knockdown of miR-485-5p as well as *BACE1*-antisense over-expression can prevent the miRNA-induced BACE1 suppression. We found that the expression of *BACE1*-antisense as well as miR-485-5p are dysregulated in RNA samples from Alzheimer's disease subjects compared to control individuals.

**Conclusions:**

Our data demonstrate an interface between two distinct groups of regulatory RNAs in the computation of *BACE1 *gene expression. Moreover, bioinformatics analyses revealed a theoretical basis for many other potential interactions between natural antisense transcripts and miRNAs at the binding sites of the latter.

## Background

Recent transcriptomic efforts have revealed surprisingly large numbers of non-protein-coding RNAs (ncRNAs) in mammalian genomes [1-4]. Classes of ncRNAs include small ncRNAs, such as microRNAs (miRNAs) and small nucleolar RNAs (snoRNAs), and several thousand long ncRNAs, including those that form complex interleaved and overlapping patterns with coding transcripts [5-7]. Natural antisense transcripts (NATs) are endogenous RNA molecules transcribed from the opposite strand of other protein-coding or non-protein-coding (sense) genes. A large-scale cDNA sequencing effort, conducted by the FANTOM-3 consortium, confirmed and greatly extended the existence of large numbers of NATs [8]. At least 1,000 pairs of sense-antisense transcripts were found well conserved between mouse and human [9]. Recently, we have identified and characterized in detail one sense-antisense pair, *BACE1 *(beta-secretase-1) and its antisense partner *BACE1*-antisense (*BACE1*-AS), and demonstrated a critical role of this non-protein-coding NAT in Alzheimer's disease [10]. Here, we report evidence that a miRNA, miR-485-5p, is involved in BACE1 post-transcriptional regulation. Together with *BACE1*-AS, miR-485-5p has the potential to participate in a ncRNA network that serves to fine-tune BACE1 protein output in the nervous system.

The mechanisms by which NATs regulate gene expression are largely unknown. The natural antisense transcript for *HIF-1α *(hypoxia inducible factor-1α) destabilizes one splice variant of *HIF *mRNA and shifts the balance in favour of the other variant [11,12]. Destabilization of one splice variant takes place by exposing the AU-rich elements in *HIF-1α *mRNA following antisense binding to its 3' UTR [11,13,14]. By contrast, stabilization of mRNA by covering the AU-rich element has been suggested for an antisense transcript of the *Bcl-2*/*IgH *hybrid gene [15]. We previously demonstrated that *BACE1*-AS enhances the stability of the *BACE1 *sense transcript [10]. Here we show that *BACE1*-AS prevents miRNA-induced translational repression and mRNA decay of *BACE1 *mRNA by 'masking' the binding site for miR-485-5p. We observed that *BACE1*-AS and miR-485-5p ncRNAs compete for binding to the sixth exonic region of *BACE1 *mRNA. Covering the miR-485-5p miRNA-binding site by *BACE1*-AS transcripts might eliminate miRNA-induced translational repression and *BACE1 *mRNA decay. Considering the reported effects of miRNAs on mRNA stability [16], cytoplasmic sense-antisense RNA duplex formation can potentially inhibit the interactions between miR-485-5p and *BACE1 *mRNA to explain, in part, the enhancement of *BACE1 *mRNA stability by *BACE1*-AS transcripts.

## Results

### *BACE1*-AS masks the binding site of miR-485-5p

**Figure 1:**
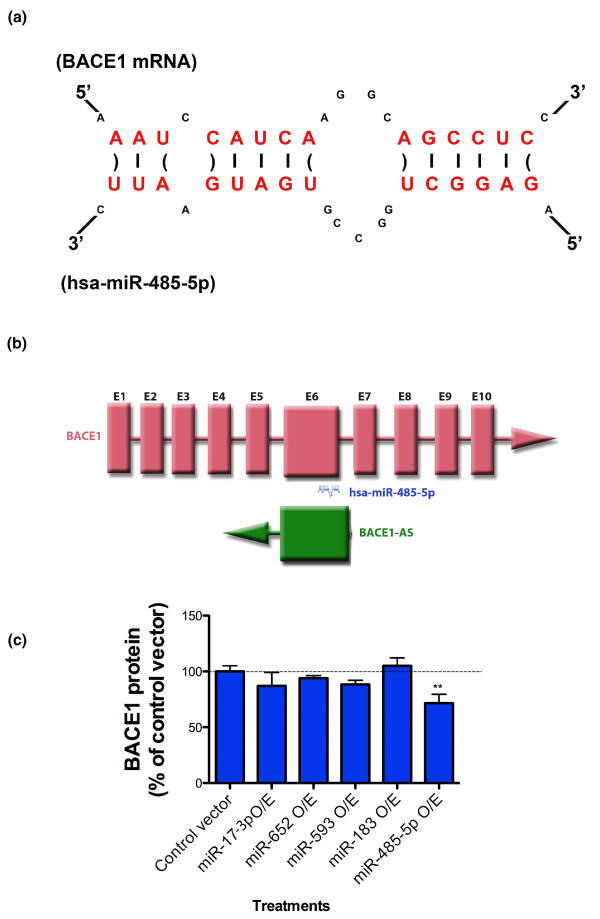
***BACE1*-AS and miR-485-5p competing for the same binding site in *BACE1 *mRNA**. **(a) **Sequence information of miR-485-5p and its target site in *BACE1 *mRNA. Binding site in *BACE1 *mRNA has a strong affinity to the miR-485-5p (free energy = -26.3 using Microinspector; -31.5 using RNA22; -22.8 using miRacle). The predicted target sequence AAGCTGTAGTCAAATCCATCAAGGCAGCCTCC is found within exon 6 of *BACE1*. **(b) **The schematic shows the predicted target site for miR-485-5p, the *BACE1*-AS transcript and their relation to *BACE1 *mRNA. The binding site for miR-485-5p is located in the overlapping region of *BACE1 *and *BACE1*-AS. *BACE1 *exons are marked as E1 to E10. Both *BACE1*-AS and miR-485-5p have the potential to bind to exon 6 (E6) of *BACE1 *mRNA. **(c) **Over-expression of miR485-5p, but not vectors that over-express miR-17-3p, miR-652, miR-593, or miR-183, nor control empty vector, leads to BACE1 protein reduction by about 30% (**P-value < 0.01). Each treatment consists of 24 repeats and error bars represent standard error of means. In this experiment, the miRNA-binding site was not artificially engineered; rather, it is located in its usual place in the open reading frame of the *BACE1 *transcript. BACE1 protein level was measured by DiscoverRx technology.

miRNAs constitute a class of noncoding regulatory RNA that functions by binding to target RNAs [17]. We have conducted a bioinformatics search for miRNA binding sites in *BACE1 *mRNA and predicted the presence of a binding site for miR-485-5p in the sixth exon of *BACE1 *mRNA. Previously we showed that the same region of *BACE1 *mRNA may interact with a natural antisense transcript, *BACE1*-AS, and that there is potential for sense-antisense RNA duplex formation (Figure 1a, b). Considering RNA duplex formation between *BACE1 *and *BACE1*-AS, we postulated that an additional regulatory function of *BACE1*-AS may be 'masking' the miR-485-5p binding site and thereby blocking the inhibitory effects of this miRNA on BACE1 translation and mRNA decay (Figure 1a, b). Some other miRNA target sites were found in the overlapping region of the *BACE1 *mRNA; however, the assigned score and binding energy were not sufficient to be considered as strong candidates. We selected a number of these miRNAs, including miR-17-3p, miR-652, miR-593 and miR-183, and over-expressed these in our cellular model, with a beta-galactosidase reporter assay corresponding to BACE1 protein as a read-out (see below). We found that, unlike miR-485-5p, these miRNAs were not able to alter BACE1 protein concentrations (Figure 1c). Although these miRNAs did not pass our validation studies, we cannot completely exclude potential interactions between these miRNAs and *BACE1 *or *BACE1*-AS transcripts.

### Target site validation by luciferase constructs

**Figure 2:**
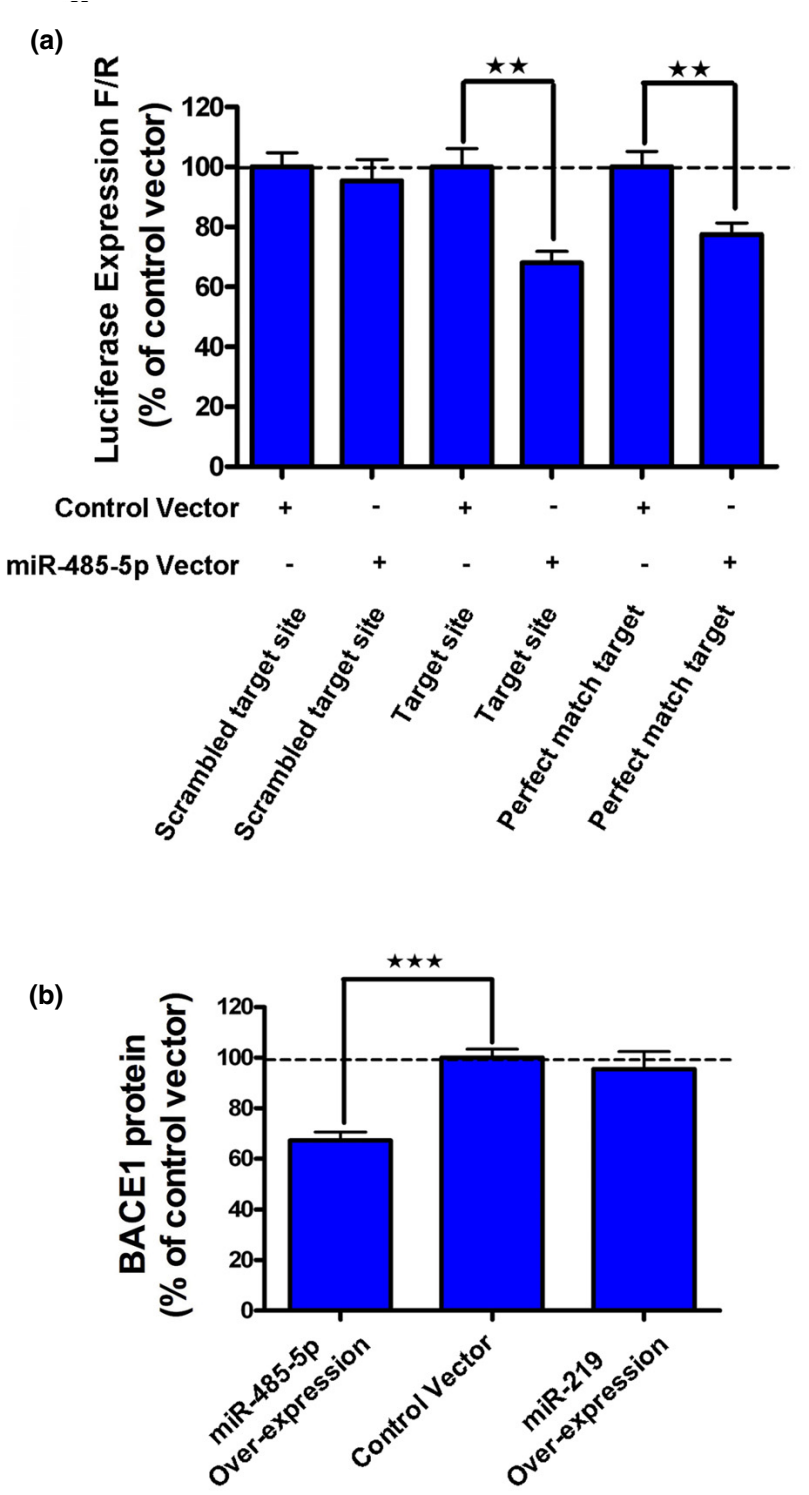
**Validation of the miR-485-5p binding site in the *BACE1 *transcript**. **(a) **The presence of the miR-485-5p target site in the 3' UTR of firefly luciferase is sufficient for depleting luciferase expression by 30%, equally effective as a perfect match positive control. The scrambled target site did not show any effect (**P-value < 0.01). This experiment was performed in HEK293T cells and each treatment consisted of 24 biological repeats; error bars represent standard error of means (SEM). **(b) **Over-expression of miR485-5p, but not control miRNA (miR-219) or empty vector, leads to BACE1 protein reduction by about 30% (***P-value < 0.001). Each treatment consists of 32 biological repeats and error bars represent SEM. In this experiment, the miRNA-binding site was not artificially engineered; rather, it is located in its usual place in the open reading frame of the *BACE1 *transcript. BACE1 protein level was measured by DiscoverRx technology.

To validate the predicted miR-485-5p target site in *BACE1 *mRNA, we engineered a miR-485-5p target site downstream of a luciferase reporter gene. This sequence corresponds to the predicted target site of miR-485-5p on *BACE1 *mRNA. We found that the presence of this target site is sufficient for luciferase reporter protein reduction upon miR-485-5p over-expression (Figure 2a). We also constructed a luciferase reporter with either full complementary or mismatch target sites for miR-485-5p as positive and negative controls, respectively. Each one of these three luciferase constructs was transfected into HEK293T cells in the presence of miR-485-5p over-expressing or empty control vectors. We observed significant down-regulation of luciferase in the construct with miR-485-5p target sites in the presence of a miR-485-5p over-expressing vector. Our results indicate that the presence of our predicted miR-485-5p target site is sufficient for miRNA binding, suggesting the possibility of *in vivo *interactions between miR-485-5p and its cognate binding site in the sixth exonic region of the *BACE1 *mRNA.

### Validation of a binding site in the coding region

Although luciferase reporters are extensively utilized as a validation tool for miRNA targets, these constructs have limitations for evaluating binding sites located in the coding region. To create a construct that resembles an *in vivo *setting, we cloned full-length *BACE1 *cDNA, excluding the 3' UTR, into a ProLabel C3 expression vector (DiscoveRx). The ProLabel C3 vector, upon transfection into mammalian cells, expresses *BACE1 *mRNA and protein with a small fusion tag. The protein tag is then used for the detection of protein synthesis utilizing the enzyme fragment complementation (EFC) system. Next, we examined the effect of miR-485-5p over-expression on BACE1 protein in HEK293T C3 cells using enzyme complementation protein quantification technology (DiscoveRx). We found that miR-485-5p over-expression causes a reduction in BACE1 protein concentrations (Figure 2b). Our results indicate that miRNA-binding sites in the coding parts of mRNAs may still be functional and further suggest the possibility of *in vivo *interactions between miR-485-5p and mature *BACE1 *mRNA.

### Locked nucleic acid-antimiR blocks miR-485-5p effects on BACE1 protein

**Figure 3:**
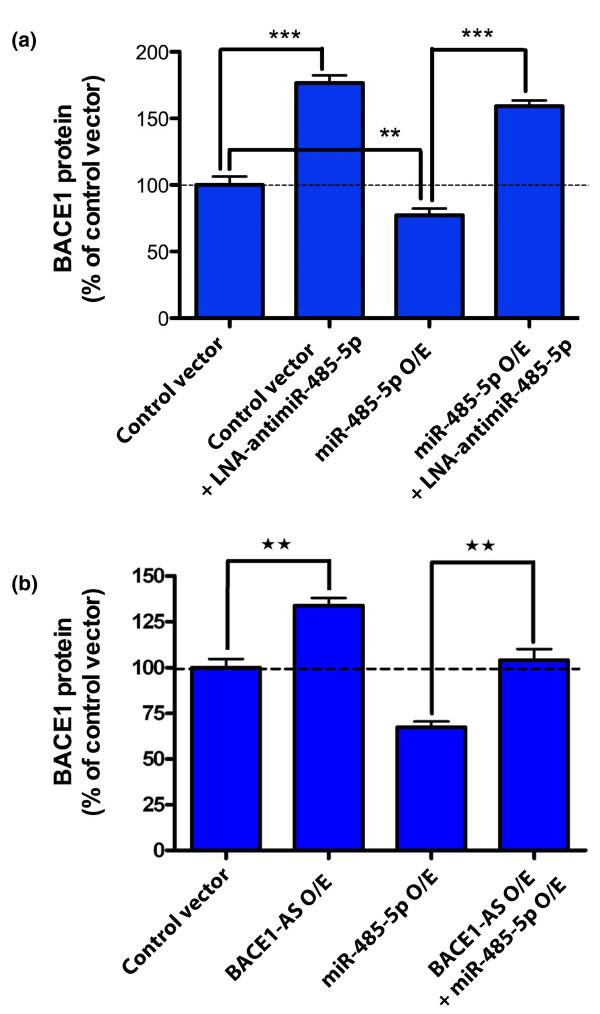
***BACE1*-AS masks the binding site for miR485-5p on *BACE1 *mRNA**. **(a) **Over-expression (O/E) of miR485-5p significantly reduces BACE1 protein levels in HEK293T C3 cells. BACE1 protein was measured by EFC protein quantification technology (DiscoveRx). LNA-antimir-485-5p, a sequence complementary to the mature miR-485-5p, blocks the effect of miRNA on BACE1 protein expression. LNA-antimiR-485 increases BACE1 protein levels by blocking endogenous miR-485-5p. Each treatment consists of 24 biological repeats and error bars represent standard error of means (SEM; **P-value < 0.01 and ***P-value < 0.001). **(b) **Simultaneous over-expression of miR-485-5p and *BACE1*-AS can effectively block the observed BACE1 protein reduction caused by miR-485-5p alone. This indicates that the two ncRNAs can compete for the same binding site on *BACE1 *mRNA. Each treatment consists of 24 biological repeats and error bars represent SEM (**P-value < 0.01).

To test the specificity of the reduction of BACE1 and to further validate the miR-485-5p target site in the coding region of *BACE1*, we applied a synthetic locked nucleic acid (LNA)-antimiR molecule to block the miRNA binding. Such antimiRs are synthetic, LNA-modified RNA molecules with sequence complementary to the mature miRNA. As expected, over-expression of miR-485-5p reduced the BACE1 protein levels and this reduction was blocked by application of LNA-antimiR-485-5p (Figure 3a). We observed that the LNA-antimiR increased BACE1 protein levels in our EFC reporter assay, which may possibly be explained by inhibition of endogenous miR-485-5p. The reversal of mir-485-5p-mediated BACE1 protein reduction by LNA-antimiR indicates the specificity of the miRNA effect and further validates the miR-485-5p binding site in the coding region of *BACE1 *mRNA.

### Noncoding RNAs compete for binding sites

Considering that *BACE1*-AS and miR-485-5p share potential binding sites in the *BACE1 *mRNA, we aimed to check the possible counteraction of these two ncRNA transcripts. If these two ncRNAs can compete for binding sites, then simultaneous over-expression should block the effect of miR-485-5p. Indeed, we noted that over-expression of *BACE1*-AS eliminated the effects of miR-485-5p and returned the BACE1 protein to basal levels (Figure 3b). We previously showed that over-expression of *BACE1*-AS caused an increase in BACE1 protein level and we were able to reproduce these data, using our *in vitro *EFC assay. On the other hand, we observed that miR-485-5p over-expression reduced the BACE1 protein levels. Simultaneous over-expression of both *BACE1*-AS and miR-485-5p returned the BACE1 protein level to the basal level. These data imply that miR-485-5p and *BACE1*-AS may compete for binding to *BACE1 *mRNA and support the novel regulatory role of masking a miRNA-binding site in *BACE1 *by the non-coding *BACE1*-AS transcript. This proposed miRNA masking effect is in concert with the concordant antisense regulatory action of *BACE1*-AS.

### Expression of miR-485-5p, *BACE1 *and *BACE1*-AS in different brain regions

**Figure 4:**
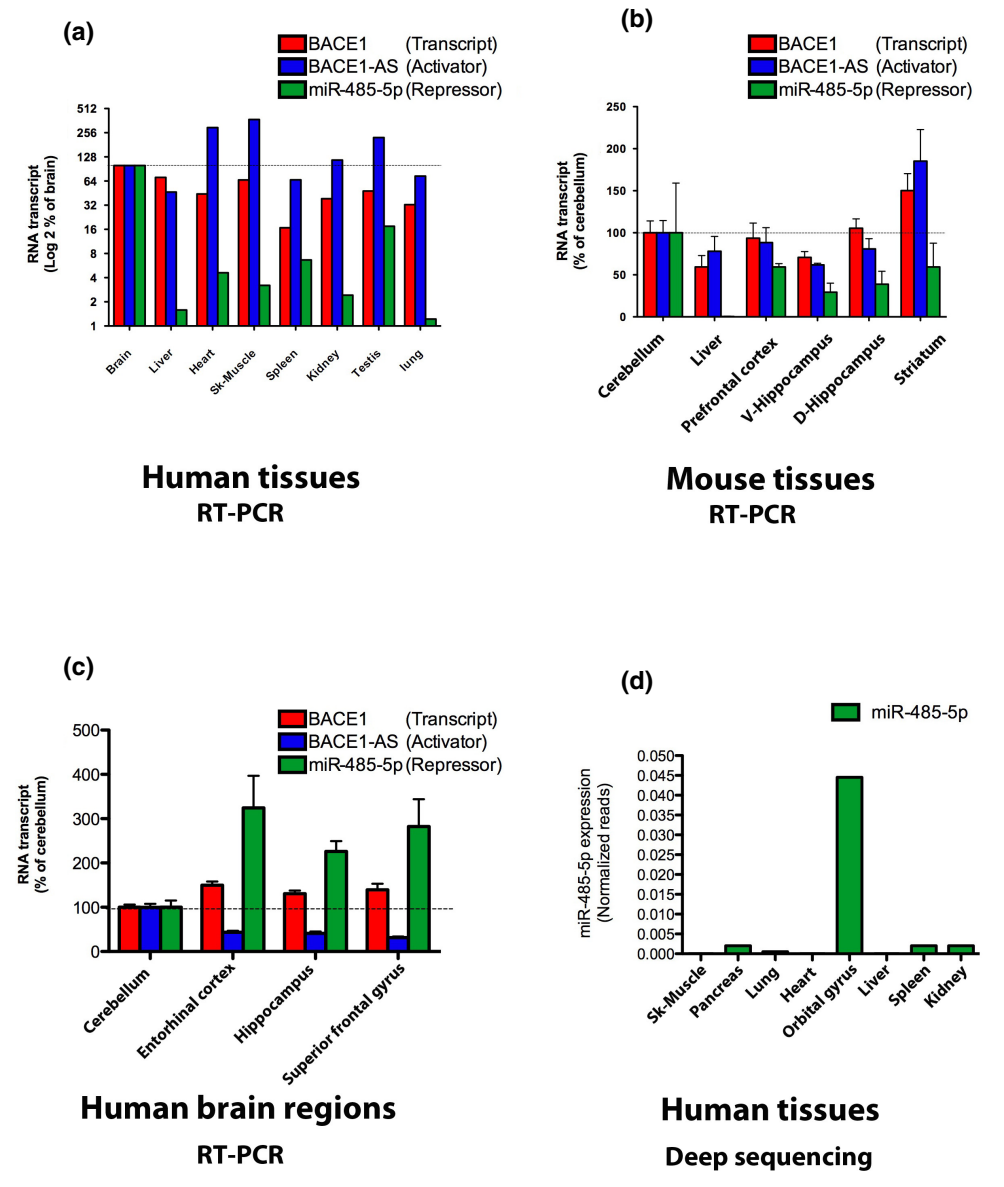
**Expression of *BACE1*, *BACE1*-AS and miR-485-5p in different brain regions**. **(a) **Expression of miR-485-5p, *BACE1 *and *BACE1*-AS were measured in a commercially available panel of human tissues (n = 1), including brain, liver, heart, skeletal (Sk) muscle, spleen, kidney, testis and lung, by RT-PCR. Whole brain RNA shows a much higher abundance of miR-485-5p (y-axis is log2% of brain). *BACE1 *mRNA was ubiquitously expressed, with the highest expression in brain. *BACE1*-AS transcript was expressed in all tissues, but relatively higher in brain, heart, skeletal muscle and testis. **(b) **Expression of miR-485-5p, *BACE1 *and *BACE1*-AS transcripts were measured in several mouse brain region as well as mouse liver (n = 3). miR-485-5p is readily present in various brain regions, but it is not evenly distributed in all regions tested. *BACE1 *and *BACE1*-AS transcripts are also highly expressed in all brain regions that are affected by Alzheimer's disease pathologies. **(c) **Expression of miR-485-5p, *BACE1 *and *BACE1*-AS transcripts were measured in four human brain regions. RNA originated from cerebellum (18 subjects), entorhinal cortex (8 subjects), hippocampus (12 subjects) and superior frontal gyrus (18 subjects). miR-485-5p was two- to four-fold higher in entorhinal cortex, hippocampus and superior frontal gyrus compared to cerebellum. *BACE1*-AS transcript was expressed two- to three-fold lower in entorhinal cortex, hippocampus and superior frontal gyrus compared to cerebellum. *BACE1 *mRNA is almost equally distributed in all four regions. **(d) **The small RNA fraction from two individuals for each of eight tissues (with the exception of the kidney, which had only one sample) was used for high-throughput short read sequencing. After alignment of reads to the human genome, the reads corresponding to miR-485-5p were identified and normalized to the total number of reads. There was significantly higher abundance of miR-485-5p in the orbital gyrus of the brain compared to skeletal muscle, pancreas, lung, heart, liver, spleen and kidney.

To confirm the expression of *BACE1*, *BACE1*-AS and miR-485-5p in brain and other human tissues, we performed real-time PCR (RT-PCR) on RNA samples from human and mouse. We observed that miR-485-5p is present and significantly higher in brain compared to other regions (Figure 4a). *BACE1 *and *BACE1*-AS transcripts showed ubiquitous expression patterns with minimal variation among different tissues. Similar results were observed in mouse tissues and various regions of mouse brain showed high expression of miR-485-5p, *BACE1 *and *BACE1*-AS transcripts (Figure 4b). The high concentration of miR-485-5p and *BACE1*-AS in the brain regions suggests the likelihood of their functional interaction with the *BACE1 *mRNA target site, and involvement in BACE1 regulation.

Next, we examined the expression of *BACE1*, *BACE1*-AS and miR-485-5p in four brain regions from human control subjects (Figure 4c). These RNA samples originated from post-mortem brains of 35 elderly individuals with an average age of 72.3 years (range 53 to 91 years) who had passed away from causes other than Alzheimer's disease. Although not all regions were available from all cases, we examined RNAs from cerebellum (18 subjects), entorhinal cortex (8 subjects), hippocampus (12 subjects) and superior frontal gyrus (18 subjects). Unlike *BACE1*-AS, miR-485-5p was two- to four-fold higher in entorhinal cortex, hippocampus and superior frontal gyrus compared to cerebellum. *BACE1*-AS transcript was expressed two- to three-fold lower in similar regions compared to cerebellum. It is worth noting that the transcript expression data represent the relative quantity of each RNA transcript to that of reference tissue (brain in Figure 4a, and cerebellum in Figure 4b, c). Therefore, these data do not directly support the conclusion that expression of miR-485-5p represses *BACE1 *transcript levels and that *BACE1*-AS expression enhances *BACE1 *transcript levels. Nevertheless, the relatively high expression of miR-485-5p and *BACE1*-AS in brain regions that are affected by Alzheimer's disease pathology may suggest a role for these ncRNAs in Alzheimer's disease-related pathogenesis.

### miR-485-5p expression as studied by high-throughput sequencing

We also examined the abundance of miR-485-5p in various human tissues by next generation sequencing of the small RNA fraction, using the Illumina Genome analyzer. Two individuals were used for sequence profiling of the small RNA fraction and identification of known miRNAs from a set of eight tissues. We found that the number of normalized reads for miR-485-5p was significantly higher in the orbital gyrus from brain compared to skeletal muscle, pancreas, lung, heart, liver, spleen and kidney (Figure 4d). The raw read count for miR-485-5p differed between the two individuals as follows: pancreas, 1.5%; lung, 1.6%; skeletal muscle, 1.5%; heart, 1.25%; brain, 1.4%; liver, 3.5%; spleen, 8.6%; and kidney (only one sample). The normalized reads from deep sequencing experiments provide absolute quantities, in contrast to relative quantity values obtained from RT-PCR methods. Therefore, the high expression of miR-485-5p in orbital gyrus of brain revealed by deep sequencing data confirms our RT-PCR findings. Moreover, the substantial abundance of miR-485-5p in the brain, compared to other tissues, suggests a neuronal-related function, and by reason of co-expression, increases the likelihood of involvement in *BACE1 *regulation.

### Expression of *BACE1*, *BACE1*-AS and miR-485-5p in Alzheimer's disease

**Figure 5:**
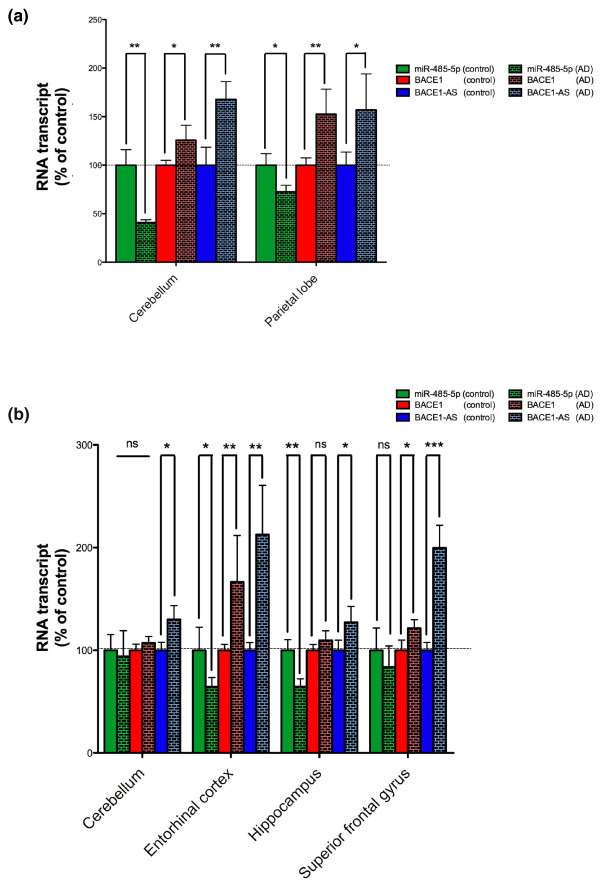
**Expression of *BACE1*, *BACE1*-AS and miR-485-5p in Alzheimer's disease-affected individuals**. **(a) **Expression of *BACE1*, *BACE1*-AS and miR-485-5p transcripts were measured in parietal lobe and cerebellum of five subjects with Alzheimer's disease and five normal elderly individuals. miR-485-5p was down-regulated by 30% in parietal lobe and 60% in cerebellum of Alzheimer's disease patients compared to control individuals. *BACE1*-AS as well as *BACE1 *transcripts were up-regulated in both cerebellum and parietal lobe (unpaired *t*-test with Welch's correction: ns = not significant; *P-value < 0.05; **P-value < 0.01; ***P-value < 0.001). **(b) **Expression of *BACE1*, *BACE1*-AS and miR-485-5p transcripts were measured in four regions of the brain of 35 Alzheimer's disease patients and 35 control individuals. Not all regions were available from all cases; a total of 120 RNA samples from superior frontal gyrus, entorhinal cortex, hippocampus and cerebellum were tested. miR-485-5p was significantly down-regulated in entorhinal cortex and hippocampus of Alzheimer's disease subjects, but not altered in cerebellum nor in superior frontal gyrus. *BACE1*-AS, and to a lesser degree *BACE1*, transcripts were up-regulated in all four regions. However, the increase in *BACE1 *mRNA was not statistically significant in cerebellum and hippocampus (unpaired *t*-test with Welch's correction: ns = not significant; *P-value < 0.05; **P-value < 0.01; ***P-value < 0.001)

We previously showed that the *BACE1*-AS transcript is significantly up-regulated in several brain regions of subjects with Alzheimer's disease. We measured the expression of *BACE1*, *BACE1*-AS and miR-485-5p in two different sets of RNA samples from control subjects and individuals with Alzheimer's disease. Initially, we examined the parietal lobe and cerebellum of 5 subjects with Alzheimer's disease and 5 normal elderly individuals (20 samples total). *BACE1*-AS, and to a lesser degree *BACE1*, transcripts were up-regulated in Alzheimer's disease patients compared to control individuals and miR-485-5p was down-regulated by 30% in parietal lobe and close to 60% in cerebellum of Alzheimer's disease patients (Figure 5a).

We have also examined a second set of RNA samples from 35 Alzheimer's disease and 35 control individuals (Figure 5b). Although not all regions were available from all cases, we examined RNA from cerebellum (18 control and 23 Alzheimer's disease subjects), entorhinal cortex (8 control and 11 Alzheimer's disease subjects), hippocampus (12 control and 12 Alzheimer's disease subjects) and superior frontal gyrus (18 control and 18 Alzheimer's disease subjects). Consistent with our previous work, *BACE1*-AS transcript concentrations were up-regulated in all four regions tested. To a lesser degree, *BACE1 *transcripts were up-regulated in entorhinal cortex as well as in superior frontal gyrus. On the other hand, miR-485-5p was significantly reduced in entorhinal cortex and hippocampus. However, miR-485-5p was not significantly altered in cerebellum and superior frontal gyrus (Figure 5b). The difference between miR-485-5p expressions in cerebellum of the two sets of RNA samples can conceivably be explained by the relatively high variability among human samples. Considering the increased level of *BACE1*-AS and reduction of miR-485-5p in the brain of Alzheimer's disease subjects, we postulated that dysregulation of these two ncRNAs might cause increases in *BACE1 *mRNA as well as the removal of the miRNA brake on BACE1 mRNA and protein expression.

### miRNA binding site enrichment in non-overlapping regions of sense-antisense pairs

**Figure 6:**
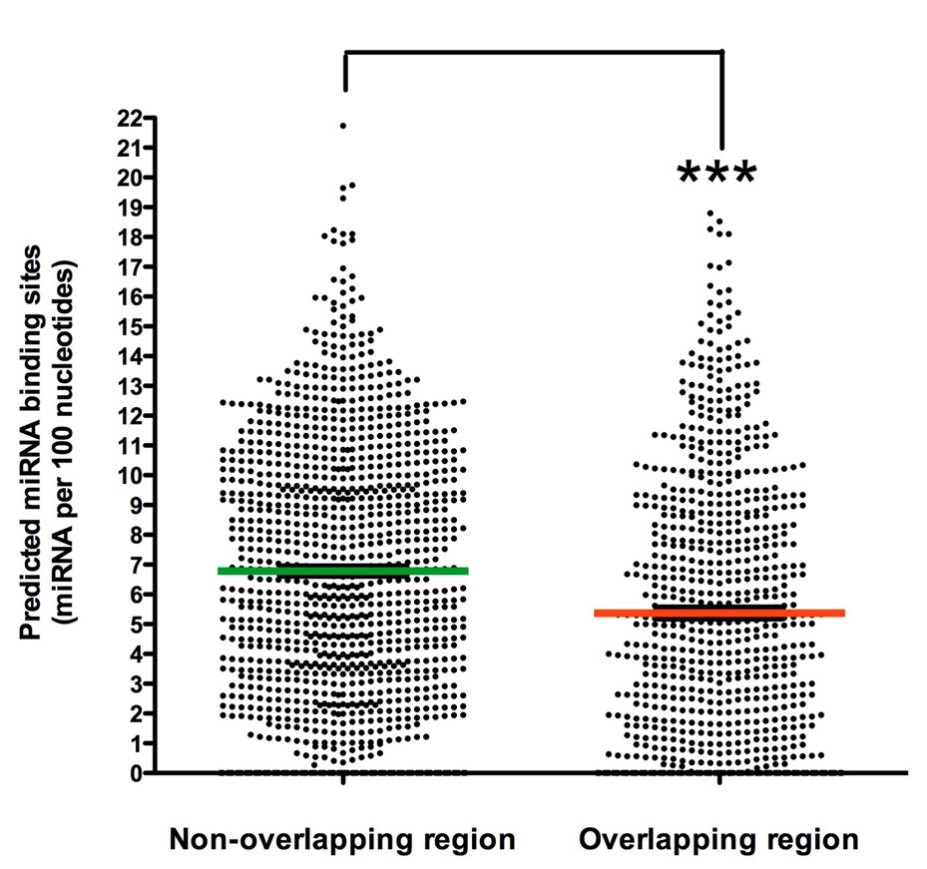
**Distribution of miRNA binding sites in sense-antisense RNA transcripts**. Predicted miRNA binding sites were counted in 391 human sense-antisense pairs. The total number of predicted miRNAs per 100 nucleotides of overlapping and non-overlapping regions of each sense-antisense pair is depicted. The miRNA binding sites within pairing and non-pairing regions have a median value of 4.78 and 6.49 miRNAs per 100 nucleotides, respectively. On average (mean), each 100 nucleotides of overlapping region have 5.21 miRNA binding sites, while each 100 nucleotides of non-overlapping region have 7.07 miRNA binding sites. The difference seen in miRNA numbers within the sense-antisense pairing regions and non-pairing regions is statistically significant (***P-value < 0.0001, Wilcoxon two-sided test).

Our data suggest an interaction between two classes of ncRNAs in the regulation of *BACE1 *gene expression. We sought to determine the extent of this computational regulatory mechanism as a general theme in the human genome. We selected a set of evolutionarily conserved sense-antisense pairs, previously published as complex loci in human and mouse genomes [9]. Predicted miRNA binding sites within pairing (sense-antisense overlapping) regions and non-pairing (non-overlapping) regions were counted and are listed in Additional file 1. In summary, among 894 sense-antisense pairs included in this study, 391 (43.7%) contain a sense-antisense overlapping region equal to or more than 25 nucleotides, which were selected for further miRNA binding-site scanning. A total of 18,704 predicted miRNA binding sites were identified in the sense-antisense pairing regions, spanning 358,663 nucleotides. In non-pairing regions, 111,192 miRNA binding sites were predicted across 1,570,606 nucleotides. After normalization of the predicted miRNA targets over sequence lengths, the miRNA binding sites within sense-antisense pairing regions ranged from 0 to 0.18790 miRNAs per nucleotide, with an interquartile range of 0.01398 to 0.08620, and a median value of 0.04780 miRNAs per nucleotide. The miRNA binding sites within non-pairing regions range from 0 to 0.2173, with an interquartile range of 0.03350 to 0.10020, and median value of 0.06490 miRNAs per nucleotide. There are more predicted miRNA binding sites in non-overlapping regions of the sense-antisense pairs compared to overlapping regions (*P*-value < 0.0001, Wilcoxon test). On average (mean), each 100 nucleotides of overlapping region have 5.21 miRNA binding sites, while each 100 nucleotides of non-overlapping region have 7.07 miRNA binding sites (Figure 6). This result was corroborated by our randomization test (*P*-value < 0.0001), in which 1,000 Monte Carlo randomizations with shuffled sequences were carried out. Our result indicates an evolutionary selection against miRNA binding to the pairing region. These findings suggest that overlapping regions between sense and antisense RNA transcripts are functional regulatory elements *per se*, and that sense-antisense RNA duplex formation may prevent miRNA binding. Therefore, there might be a selection to avoid a clash of two regulatory elements in one particular region.

## Discussion

Normal physiological levels of BACE1 protein are essential for proper cognitive, emotional and synaptic function [18], and for myelination of peripheral nerves [19,20]. On the other hand, elevation of BACE1 protein might cause overproduction of amyloid peptides, such as amyloid-β 1-42 (Aβ1-42) and Aβ1-40. Imbalance between production and clearance of Aβ1-42 could potentially lead to the cascade of amyloid precursor protein cleavage, amyloid plaque formation, and the synaptic disruption characteristic of early Alzheimer's disease. The levels of BACE1 therefore require tight regulation to maintain a narrow window between essential and pathological expression of BACE1. Sequential cleavage of amyloid precursor protein by BACE-1 and γ-secretase represents a central event in Alzheimer's disease pathophysiology, and both proteases serve as potential targets for development of novel therapeutics for Alzheimer's disease [21]. Thus, understanding the mechanisms of BACE1 regulation may reveal important insights into the etiology of Alzheimer's disease, and also facilitate the development of novel therapeutics and/or biomarkers of the disease [22-25].

We have previously demonstrated that *BACE1*-AS enhances the stability of the *BACE1 *sense transcript [10]. In this study, we show an additional, synergistic mechanism by which *BACE1*-AS regulates its sense partner, namely by preventing miRNA-induced mRNA decay and translational repression. Specifically, miR-485-5p and *BACE1*-AS likely share a common binding site in the sixth exon of the *BACE1 *mRNA transcript. Therefore, interactions between either one of these two ncRNAs and *BACE1 *mRNA would establish a finely tuned regulation of BACE1 protein production. Destabilization of mRNA after miRNA binding has been previously suggested [16]. We hypothesized that one mechanism by which *BACE1*-AS regulates *BACE1 *mRNA stability involves the 'masking' of a miR-485-5p binding site. Hence, the translational repression and destabilization of *BACE1 *mRNA by miR-485-5p is less likely to occur in the presence of the *BACE1*-AS transcript. Nonetheless, both proposed actions of *BACE1*-AS, promoting target mRNA stability by duplex formation and inhibiting miRNA-induced mRNA decay and translational repression, serve to elevate BACE1 concentrations.

Alzheimer's disease patients demonstrate increased expression of *BACE1 *mRNA and generation of Aβ1-42 compared with unaffected controls [26-30]. Our data suggest that *BACE1*-AS and miR-485-5p are both highly expressed in the nervous system. The dysregulation of these two ncRNA transcripts may induce Alzheimer's disease-related BACE1 upregulation by stabilizing the *BACE1 *transcript and by blocking miRNA-induced translational repression. Interplay between these ncRNAs might be crucial for neuronal cells to maintain a precise physiological BACE1 homeostasis involving post-transcriptional regulatory mechanisms.

Disruption of miRNA binding sites by the presence of SNPs in the 3' UTRs of mammalian genes has been clearly documented [31-35]. Variants in the binding site for miR-189 in the 3' UTR region of the *SLITRK1 *gene are associated with Tourette's syndrome [35]. Synonymous mutations in regulatory regions of mRNAs could create or destroy a putative miRNA binding site, therefore changing the protein output of specific transcripts [31]. The SNP in the 3' UTR of the myostatin gene (*GDF8*) that creates a target site for miR-1 and miR-206 contributes to the muscular hypertrophy of Texel sheep [31]. Additionally, the variant allele in a *KRAS *mRNA, associated with a *Let-7 *miRNA complementary site, is significantly linked with an increased risk for lung carcinoma [32]. A functional SNP at a miRNA binding site (miRSNP) in the 3' UTR of dihydrofolate reductase interferes with miR-24 function and leads to dihydrofolate reductase over-expression and methotrexate resistance [33,34]. These examples point to the fact that any interference between miRNAs and their binding sites would have regulatory consequences. We argue here that cytoplasmic natural antisense transcripts bind to sense mRNA and 'mask' miRNA binding sites. We think that the cytoplasmic sense-antisense RNA duplex formation is transient and reversible, as a stress response would require; therefore, interactions between sense mRNA, antisense transcript and miRNA might determine the level of protein production. In line with this hypothesis, we present evidence to show *in vitro *competition between miR-485-5p and *BACE1*-AS for binding to *BACE1 *mRNA. This novel masking function for the antisense RNA may apply to many other natural antisense transcripts.

The effect of miR-485-5p on *BACE1 *may demonstrate a non-canonical miRNA target site in the coding region of a mammalian mRNA, overlapping with the site of sense-antisense duplex formation. In contrast with plant miRNAs, most animal miRNAs are predicted to have their binding site in 3' UTR of target mRNA [36]. Although most web prediction tools for miRNA binding sites are designed to search only for 3' UTR regions of transcripts, there is no evidence against miRNA binding to the coding region. Binding of miRNA to the coding region of mRNAs, or even the 5' UTR, has been shown in plants and recently in animals [37-40]. Our results further suggest that miRNA association with any position on a target mRNA is mechanistically sufficient for binding.

In our bioinformatics study, we showed that miRNAs are predicted to target the non-overlapping region of sense-antisense RNA transcripts, outside of the genomic regions that have the potential to form sense-antisense duplex formation. This strategy might be beneficial from an evolutionary point of view, enabling the antisense sequences and the miRNAs to exert fine-tuned regulatory roles through targeting different sites on the same target mRNAs. The fact that there are fewer predicted miRNA binding sites in the overlapping region of natural antisense transcripts suggests that gene regulation, for both RNA species, takes place by ncRNA-mRNA nucleotide complementarities, and further suggests that both groups are functional regulatory elements. In this context, competition between these two regulatory elements, as in the case of *BACE1*-AS and miR-485-5p, are exceptions that would allow a more complex type of regulation. This complex regulatory architecture, combined with its evolutionary conservation, suggests a profound biological importance for the *BACE1*-AS-mediated stress response, including a biological function for the transient increase in Aβ levels that occur as a result of the regulatory action of these ncRNAs.

In fact, the stress response of the *BACE1 *sense-antisense locus involves additional elements of complexity [10]. In contrast to the *BACE1 *sense transcript, the *BACE1*-AS transcript shows a pronounced nuclear enrichment pattern, similar to other nuclear-enriched ncRNA transcripts [41]. We previously documented that the stress responsive *BACE1*-AS transcript shifts into the cytosol upon exposure to neuronal stress, contributing to a rapid but reversible increase of BACE1 protein, and Aβ production [10]. Emerging data suggest that miRNAs and NATs are both instrumental in a variety of stress responses [42,43]. A synergy between these two classes of ncRNAs as we have hypothesized here is an intriguing possibility that would significantly increase the regulatory power of these families of ncRNAs within the context of a larger ncRNA sensory and regulatory network [44]. These mechanisms, together with the unusual but specific response of the BACE1 system to stress, may explain some aspects of Alzheimer's disease and other neuropathologies related to chronic stress response.

Different cell stressors, such as hypoxia, re-oxygenation, oxidative stress and some pro-apoptotic factors, have long been implicated in the pathogenesis of Alzheimer's disease. These stressors are known to enhance BACE1 activity and Aβ1-42 production, which likely contributes to Alzheimer's disease pathophysiology [45,46]. Also, there is considerable evidence that Aβ1-42 itself is a potent cell stressor [47-49]. Further, Aβ1-42 enhances *BACE1 *mRNA and protein activity, and can thereby cause damage to neurons through various cell-stress-related mechanisms [47]. We have recently shown that a variety of cell stressors can increase *BACE1*-AS and *BACE1 *expression, therefore enhancing Aβ1-42 biosynthesis [10]. Since *BACE1*-AS regulates BACE1 *in vivo*, we propose that the elevation of *BACE1*-AS as a result of the actions of Alzheimer's disease-related cell stressors forms a basis of a deleterious feed-forward cycle of disease progression. This deleterious effect of *BACE1*-AS may, at least in part, come from the ability of this transcript to mask a miR-485-5p binding site. The increase in BACE1 protein by removing the negative effect of miRNAs might then contribute to enhanced Aβ1-42 formation and formation of amyloid plaques. It should be noted that Aβ1-42 accumulation in the Alzheimer's disease brain is a long-lasting and chronic process and that even small changes in BACE1 activity may lead to a significant increase in amyloid deposition over time [50,51].

## Conclusions

Our data demonstrate a potential competition between two different classes of ncRNAs. We present evidence to show that miR-485-5p and *BACE1*-AS transcripts compete for a binding site in the sixth exonic region of *BACE1 *mRNA. We show that the expression of *BACE1*-AS as well as miR-485-5p is dysregulated in RNA samples from Alzheimer's disease subjects compared to age and sex matched control individuals. Moreover, we show that over-expression of miR-485-5p and *BACE1*-AS has opposing regulatory effects on BACE1 protein expression. These data, along with our previous findings, indicate a ncRNA regulatory network exerting control over the expression of BACE1 and further provide an additional mechanism of NAT-mediated regulation of *BACE1 *mRNA. Our findings thus support the existence of ncRNA-containing regulatory networks that may be implicated in Alzheimer's disease pathophysiology.

## Materials and methods

### RT-PCR

RT-PCR was carried out with the GeneAmp 7900 machine (Applied Biosystems, Foster City, CA, USA). The primers and probe for miR-485-5p were bought from Applied Biosystems. The primers and probe for *BACE1 *and *BACE1*-AS were previously reported [10]. The PCR conditions were as follows: 50°C for 2 minutes then 95°C for 10 minutes then 40 cycles of 95°C for 15 s and 60°C for 1 minute. The results are based on cycle threshold (Ct) values. Differences between the Ct values for experimental and reference genes (Human beta-actin or U6 small RNA) were calculated as ΔΔCt.

### High-throughput sequencing

Sequencing was carried out on RNA from two individuals for eight tissues, pancreas, lung, heart, skeletal muscle, brain, liver, spleen and kidney (kidney had only one sample). Sequencing was carried out using the Illumina genome analyzer. Small RNA libraries were prepared and 36 cycle sequencing carried out according to the manufacturer's instructions. Briefly, total RNA was fractionated and the 18-35 nucleotide fraction isolated. RNA adapters were ligated to the 3' and 5' ends of the samples and used for cDNA synthesis. Libraries were sequenced on the genome analyzer (Illumina, San Diego, CA, USA) and the sequences analyzed using miRanalyzer [52]. The number of unique reads for miR-485-5p were counted for each tissue and normalized to the total number of reads. The short read sequence data were submitted to the Sequence Read Archive at the National Center for Biotechnology Information; the submission number is SRA012516.1 and is freely available.

### Cell culture and transfection

HEK293T cells were cultured in DMEM plus 10% fetal bovine serum and transfected with *BACE1*-AS, miR-485-5p, other miRNAs or empty vectors.

### Statistical analysis

All experiments were performed with at least three to six biological and technical repeats. Beta-galactosidase and luciferase studies were performed in 96-well plates with at least 24 replicates for each treatment. The data are presented in graphs as a comparison with controls, after *post hoc *test of treatment factor using main effect in two-way analysis of variance (ANOVA). Alternatively, an unpaired *t*-test with Welch correction was used to calculate significance of the differences. The significance of each treatment was calculated as a *P*-value and is reported in the legend of each figure; *P *< 0.05 was considered significant.

### Enzyme complementation assay (DiscoveRx)

ECA is a technology developed by DiscoveRx (Fremont, CA, USA) that allows the measurement of changes in protein levels. The cDNA of *BACE1 *was cloned into pCMV-ProLabel vector upstream of the ProLabel and transfected into HEK293T cells to produce a fusion protein (BACE1 and the enzyme donor fragment) expressed in a stable cell line we call C3. When the two fragments (enzyme donor and enzyme acceptor) of the β-galactosidase combine in solution, the enzyme becomes active and hydrolyzes a substrate that produces a chemiluminescent signal. The strength of this signal is proportional to the protein being produced (in this case *BACE1*). The stable cell line C3 over-expressing BACE1 was transfected with *BACE1*-AS over-expression vector, miRNA over-expression vector or control vectors and protein expression measured 48 hours later with the DiscoveRx method; data are plotted as a percentile of control vector.

## Luciferase and miR-485-5p over-expression experiments

The predicted target site of miR-485-5p on *BACE1 *mRNA was engineered into the 3' UTR of a firefly luciferase construct; as a positive control we used a perfect match to miR-485-5p, and as a negative control we used a scrambled target site inserted downstream of firefly luciferase. LNA-antimiR for miR-485-5p was purchased from Exiqon (Exiqon, Vedbaek, Denmark) and transfected at 10 nM concentration. The firefly luciferase construct along with a renilla luciferase construct (Prltk, Promega, WI, USA) and miRNA over-expression vectors were transfected into HEK293T cells in 96-well plates. Luciferase activity was assessed 48 hours later and firefly luciferase expression normalized to the renilla and graphed as a percent of controls. Alternatively, miRNA over-expression vectors (with pre-miRNA constructed into the pMSCV vector; kindly provided by Dr Corinne Lasmezas) - miR-485, miR-219 as a control or empty vector - were transfected into the HEK293T C3 cell line (ProLabel, DiscoveRx) and protein quantification was performed using DiscoveRx technology.

### Human samples

The first set of human brain samples was prepared at the USC Alzheimer's Disease Research Center, which obtained informed consent from all subjects; the USC Institutional Review Board then approved the use of the human tissues. RNA was extracted from parietal lobes and cerebellum of postmortem brains from five subjects with Alzheimer's disease and five matched controls. The average age of subjects with Alzheimer's disease was 85 years (range 75 to 92 years) and 91.8 years (90 to 95 years) for controls. The postmortem interval ranged from 3.75 to 10.1 h with a mean of 5.87 h. We treated RNA samples with DNase and purified them with RNeasy mini columns (QIAGEN Valencia, CA, USA). We prepared cDNA from 400 ng of RNA samples and used RT-PCR for relative quantification of different transcripts.

The second set of human brain samples and the tissues used for deep sequencing were prepared from rapid autopsy brain tissue and were collected by J Rogers and T Beach (Sun Health Research Institute); all enrolled subjects or legal representatives had signed a Sun Health Research Institutional Review Board-approved informed consent form allowing both clinical assessments during life and several options for brain and bodily organ donation after death. These cases included 35 autopsy confirmed cases of Alzheimer's disease with an average age of 81.8 years (range 64 to 92 years) and 35 controls with an average age of 72.3 years (range 53 to 91 years). The postmortem interval ranged from 1.25 to 5 h with a mean of 2.5 h. The average duration of disease in the subjects with Alzheimer's disease was 9.2 years. Total RNA was isolated via CsCl purification from tissue dissected from specific regions of brain. Although not all regions were available from all cases, we examined a total of 120 RNA samples from superior frontal gyrus, entorhinal cortex, hippocampus and cerebellum for *BACE1*, *BACE1*-AS and miR-485-5p expression by RT-PCR.

A panel of total RNA from human tissues was bought from Applied Biosystems and used for expression profiling of *BACE1*, *BACE1*-AS and miR-485-5p.

### Bioinformatics detection of miRNA binding sites

Published human miRNA sequences were retrieved from the miRBase database [53]. A list of 993 evolutionarily conserved natural antisense transcripts was retrieved from the previously published FANTOM-3 dataset [9]. Out of this list, 99 pairs were deleted from any analyses described in this study, as we could not verify that they indeed qualify as sense-antisense pairs. miRNAs were screened in the sense-antisense RNA overlapping and non-overlapping regions, respectively. The BLAST program [54] was used to obtain the overlapping and non-overlapping regions of each sense-antisense pair. Then, the miRanda algorithm [55] was used for miRNA binding-site predictions. The numbers of predicted miRNAs within sense-antisense pairing regions and non-pairing regions were normalized to sequence length. Wilcoxon two-sided test was computed in R to compare the difference in predicted miRNA binding sites within sense-antisense pairing and non-pairing regions. In the randomization test, all sequences derived from the pairing and non-pairing regions were shuffled and sequences were randomly selected to create two data sets to represent the simulated pairing and non-pairing region data, respectively. The numbers of sequences included in the simulated data sets were equal to those in the real data sets. Wilcoxon two-sided test was used to compare the predicted miRNAs between the simulated pairing and non-paring data sets. The randomization procedure was repeated 1,000 times. We calculated the number of times (x) that the Wilcoxon *P*-value/W values in the randomizations are smaller than those from the real data sets. The *P*-value of the randomization is x/1,000. The statistically significant difference cut-off was 0.05.

## Abbreviations

Aβ: amyloid-β; *BACE1*: beta-secretase-1; *BACE1*-AS: BACE1-antisense transcript; EFC: enzyme fragment complementation; HIF: hypoxia inducible factor; LNA: locked nucleic acid; miRNA: microRNA; NAT: natural antisense transcript; ncRNA: non-protein-coding RNA; RT-PCR: real-time PCR; SNP: single nucleotide polymorphism; UTR: untranslated region.

## Authors' contributions

MAF conceived designed and carried out the experiments, analyzed the data and drafted the manuscript. MZ carried out the Bioinformatics analysis. JH and FM participated in the design of the study and performed some of the experiments. MPVDB, MAN and MRC designed, performed and analyzed the deep sequencing data. GSL participated in experimental design and coordination. CW provided project oversight, conceived the study, and participated in its design and coordination. All authors read and approved the final manuscript.

## Supplementary Material

Additional file 1 **an Excel spreadsheet listing conserved sense-antisense pairs, their accession numbers and potential miRNA-binding sites in overlapping and non-overlapping regions**. Click here for file
